# Comparison of outcomes following the Fontan procedure between patients with previous ductus stent and aortopulmonary shunt

**DOI:** 10.1093/icvts/ivaf118

**Published:** 2025-05-21

**Authors:** Dimitrij Grozdanov, Muneaki Matsubara, Takuya Osawa, Jonas Palm, Thibault Schaeffer, Carolin Niedermaier, Nicole Piber, Paul P Heinisch, Stanimir Georgiev, Alfred Hager, Peter Ewert, Jürgen Hörer, Masamichi Ono

**Affiliations:** Department of Congenital and Pediatric Heart Surgery, German Heart Center Munich, University Hospital of Technische Universität München, Munich, Germany; Division of Congenital and Pediatric Heart Surgery, University Hospital of Munich, Ludwig-Maximilians-Universität, Munich, Germany; Europäisches Kinderherzzentrum München, Munich, Germany; Department of Congenital and Pediatric Heart Surgery, German Heart Center Munich, University Hospital of Technische Universität München, Munich, Germany; Division of Congenital and Pediatric Heart Surgery, University Hospital of Munich, Ludwig-Maximilians-Universität, Munich, Germany; Europäisches Kinderherzzentrum München, Munich, Germany; Department of Congenital and Pediatric Heart Surgery, German Heart Center Munich, University Hospital of Technische Universität München, Munich, Germany; Division of Congenital and Pediatric Heart Surgery, University Hospital of Munich, Ludwig-Maximilians-Universität, Munich, Germany; Europäisches Kinderherzzentrum München, Munich, Germany; Department of Congenital Heart Disease and Pediatric Cardiology, German Heart Center Munich, University Hospital of Technical University of Munich, Munich, Germany; Department of Congenital and Pediatric Heart Surgery, German Heart Center Munich, University Hospital of Technische Universität München, Munich, Germany; Division of Congenital and Pediatric Heart Surgery, University Hospital of Munich, Ludwig-Maximilians-Universität, Munich, Germany; Europäisches Kinderherzzentrum München, Munich, Germany; Department of Congenital and Pediatric Heart Surgery, German Heart Center Munich, University Hospital of Technische Universität München, Munich, Germany; Division of Congenital and Pediatric Heart Surgery, University Hospital of Munich, Ludwig-Maximilians-Universität, Munich, Germany; Europäisches Kinderherzzentrum München, Munich, Germany; Department of Cardiovascular Surgery, German Heart Center Munich, University Hospital of Technische Universität München, Munich, Germany; Department of Congenital and Pediatric Heart Surgery, German Heart Center Munich, University Hospital of Technische Universität München, Munich, Germany; Division of Congenital and Pediatric Heart Surgery, University Hospital of Munich, Ludwig-Maximilians-Universität, Munich, Germany; Europäisches Kinderherzzentrum München, Munich, Germany; Department of Congenital Heart Disease and Pediatric Cardiology, German Heart Center Munich, University Hospital of Technical University of Munich, Munich, Germany; Department of Congenital Heart Disease and Pediatric Cardiology, German Heart Center Munich, University Hospital of Technical University of Munich, Munich, Germany; Department of Congenital Heart Disease and Pediatric Cardiology, German Heart Center Munich, University Hospital of Technical University of Munich, Munich, Germany; Department of Congenital and Pediatric Heart Surgery, German Heart Center Munich, University Hospital of Technische Universität München, Munich, Germany; Division of Congenital and Pediatric Heart Surgery, University Hospital of Munich, Ludwig-Maximilians-Universität, Munich, Germany; Europäisches Kinderherzzentrum München, Munich, Germany; Department of Congenital and Pediatric Heart Surgery, German Heart Center Munich, University Hospital of Technische Universität München, Munich, Germany; Division of Congenital and Pediatric Heart Surgery, University Hospital of Munich, Ludwig-Maximilians-Universität, Munich, Germany; Europäisches Kinderherzzentrum München, Munich, Germany

**Keywords:** single ventricle, ductus stent, aortopulmonary shunt, pulmonary artery, Fontan procedure

## Abstract

**OBJECTIVES:**

In this study, we aimed to compare the outcome after the Fontan procedure in patients after an initial ductus stenting or a surgical aortopulmonary shunt.

**METHODS:**

We reviewed infants with single ventricle and ductal-dependent pulmonary blood flow who underwent ductus stenting or an aortopulmonary shunt between 2009 and 2022, and subsequently underwent the staged Fontan procedure.

**RESULTS:**

A total of 93 patients were included (39 ductus stenting and 54 aortopulmonary shunts). Before the Fontan procedure, pulmonary artery pressure (9 vs 9 mmHg, *P* = 0.376) and pulmonary artery index (184 vs 183 mm^2^/m^2^, *P* = 0.988) were similar between the groups. However, the incidence of venovenous collaterals was higher in patients after ductus stenting than those after aortopulmonary shunt (35.9 vs 16.7%, *P* = 0.034). Median age (1.9 vs 1.8 years, *P* = 0.493) and weight at the Fontan procedure (12 vs 11 kg, *P* = 0.596) were similar between the groups. There was no in-hospital mortality in each group. The length of the intensive care unit stay (median 5 vs 5 days, *P* = 0.542) and hospital stay (median 17 vs 14 days, *P* = 0.767) were similar between the groups. During the median follow-up of 2.5 years, one late death was observed in the ductal stenting group. Freedom from reintervention (66.6 vs 82.0%, *P* = 0.095) and from adverse events (78.6 vs 92.2%, *P* = 0.488) at 5 years were similar between the groups.

**CONCLUSIONS:**

This pilot study demonstrated comparable outcomes following the Fontan procedures between patients with single ventricle and ductal-dependent pulmonary blood flow after initial ductus stenting and those after initial aortopulmonary shunt.

## Graphical Abstract

**Figure:**
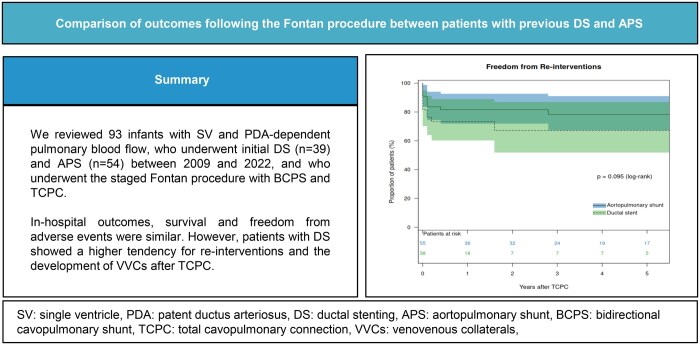

## INTRODUCTION

In neonates with single ventricle and patent ductus arteriosus (PDA)-dependent pulmonary circulation, ductal stenting (DS) emerged as an alternative to aortopulmonary shunt (APS) [1, 2]. While interventional DS has proven to be a less invasive approach than surgical APS, it carries potential disadvantages, including procedural-related complications, increased need for reintervention and impaired pulmonary artery (PA) growth [3–8]. It is well known that patients with single ventricle and pulmonary atresia are at the highest risk of death and re-interventions after APS compared to those with other anatomical types [9–12]. Therefore, DS has become a standard procedure for such patients despite these potential disadvantages. Previous studies comparing DS and APS have been mainly performed in patients with both uni- and biventricular hearts , and few studies have focused on univentricular patients only [3–8]. There is a controversy regarding the growth of PA after the initial DS and APS [5, 13–16]. Our previous study demonstrated lower left PA development at the time of stage II palliation through bidirectional cavopulmonary shunt (BCPS) in patients after DS compared to those after APS [8]. Furthermore, there is currently very limited evidence on the outcomes after the Fontan procedure in this cohort regarding different initial interventions of DS and APS.

This study compares the outcomes after extracardiac total cavopulmonary connection (TCPC) in patients who initially underwent DS versus those who received an APS, regarding preoperative condition, operative variables, hospital recovery, complications and follow-up outcomes.

## METHODS

### Ethical statement

This study was approved by the Institutional Review Board of the Technical University of Munich (approval number 2024-334-S-CB on 08 July 2024). Because of the retrospective nature of the study, the need for individual patient consent was waived. Any collection and storage of data from research participants for multiple and indefinite use is consistent with requirements outlined in the WMA Declaration of Taipei. The ethics committee approved the establishment and monitor ongoing use of databases.

### Patients and data collection

We reviewed all patients who underwent staged TCPC after DS or APS as initial palliation, performed at the German Heart Center Munich between 2009 and 2022. Infants with hypoplastic left heart syndrome and its variants who underwent the Norwood procedure were excluded from this study. Medical records were reviewed, including in-hospital and outpatient notes, echocardiography and cardiac catheterization findings. The follow-up data were regularly tracked using our institutional single ventricle database system. The PA index before BCPS and TCPC was calculated using angiography as described by Nakata and colleagues [17]. The right PA- and the left PA indices were calculated by dividing the cross-sectional area of each PA branch by the body surface area. A PA symmetry index was also calculated to evaluate the symmetry of the PA development [5]. The presence of venovenous collaterals (VVCs) was determined through the angiogram of the upper systemic veins, thoracic inferior vena cava and PAs. We defined VVCs as originating from the superior systemic venous circulation if they had an identifiable angiographic origin, supplying the other systemic or pulmonary parenchyma, and opacifying the pulmonary veins, inferior vena cava, atria or a combination of them. Angiograms in which collateral vessels were visualized but with ambiguous origin or blood flow distribution were classified as VVC negative.

### Echocardiography

Preoperative ventricular function and atrioventricular valve (AVV) function were evaluated using echocardiography. Systemic VF was qualitatively assessed, and the AVV regurgitation was graded as previously described [18].

### Adverse outcomes

The adverse events after TCPC were defined as death, protein-losing enteropathy (PLE), plastic bronchitis, thrombus formation in TCPC pathway, and New York Heart Association class III/IV.

### Operative techniques and anticoagulation

The techniques for extracardiac TCPC were previously described [19, 20]. Fenestration was not routinely performed but only in high-risk patients [15]. Because of the low cardiac output status of Fontan circulation and the presence of prosthetic material, our patients received phenprocoumon (Marcumar) with a target International Normalized Ratio (INR) between 2.0 and 3.0. Before TCPC, acetylsalicylic acid was administered in patients after APS, while clopidogrel, a platelet aggregation inhibitor, was utilized after DS.

### Statistical analysis

Categorical variables are presented as absolute numbers and percentages. A chi-squared test (observed frequency ≥ 5) and Fisher’s exact test (observed frequency < 5) were used for categorical data analysis. Continuous variables are expressed as medians with interquartile ranges (IQRs). An independent sample Student’s t-test was used to compare normally distributed variables. Levene’s test was used to differentiate between normal and non-normal distributions. The Mann–Whitney *U*-test was used for variables that were not normally distributed. The median potential follow-up time was calculated using the reverse Kaplan–Meier method, and the completeness of follow-up was assessed using both Clark’s Completeness Index (CCI) and the Simplified Person-Time (SPT) method. Survival after TCPC, freedom from re-interventions and freedom from adverse events were calculated using the Kaplan–Meier method, and the differences between the groups were determined using a log-rank test. Risk factors for reinterventions after TCPC were analysed using a Cox regression model. Patients’ main diagnoses, associated anomalies, DS or APS, pre-BCPS haemodynamic variables and PA indexes and pre-TCPC variables (age at TCPC, presence of APCs and VVCs, pre-TCPC haemodynamic variables and PA indexes) were used for the analysis. The proportional hazard assumptions were verified using Schoenfeld residuals. Those variables with a *P*-value of <0.1 in univariable analysis were considered for entry into the multivariable models. *P*-values <0.05 were considered significant. Data analysis was performed using SPSS version 28.0 for Windows (IBM, Ehningen, Germany) and R statistical software version 4.2.1. (R Foundation for Statistical Computing).

## RESULTS

### Patient characteristics

**Figure 1:**
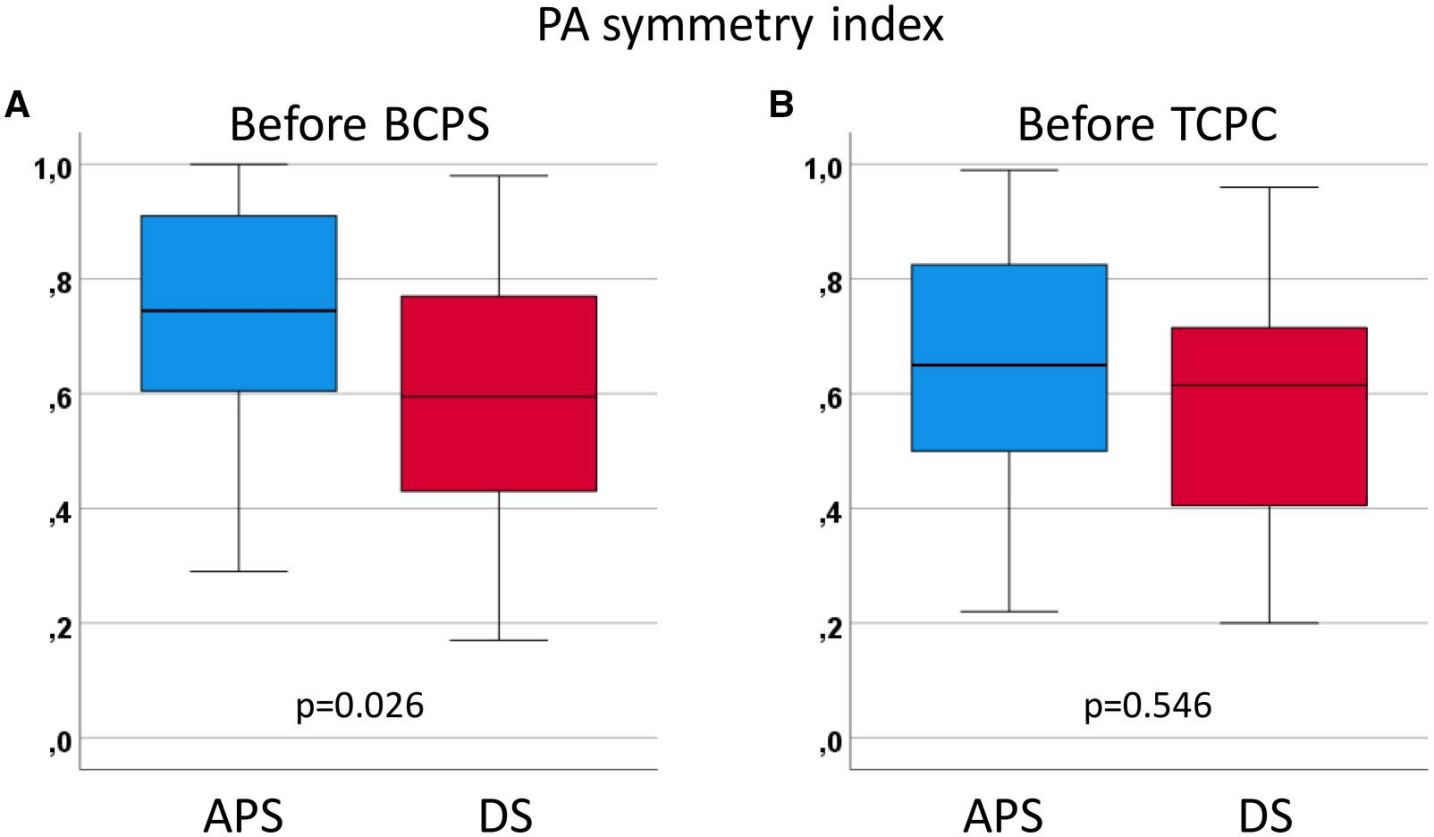
Comparison of the symmetric index between patients after DS and those after APS at the time of BCPS (**A**) and at the time of TCPC (**B**).

A total of 93 patients were identified, including 39 (42%) patients who initially underwent DS, and 54 (58%) patients who underwent APS. The flowchart of the original patient cohort is shown in Supplementary Fig. 1. There was no difference in the cumulative incidence of reaching stage III palliation (89.2 vs 87.2% at 3 years, *P* = 0.578) between the DS and the APS group. No significant difference was observed in patient characteristics between the groups (Table 1). As for the BCPS data, preoperative PAP (15 vs 14 mmHg, *P* = 0.538) and preoperative PA index (181 vs 172mm^2^/m^2^, *P* = 0.929) were similar between the groups. However, PA symmetry index was lower in the DS group than in the APS group (0.60 vs 0.75, *P* = 0.026, Fig. 1A).

**Table 1:** Patients’ characteristics and procedural variables

Variables	Total	DS	APS	*P*-value
*N* (%) or median (IQR)	*n* = 93	*n* = 39	*n* = 54	
Patients’ characteristics				
Primary diagnosis				
Tricuspid atresia	21 (22.6)	10 (25.6)	11 (20.4)	0.549
Univentricular heart	17 (18.3)	4 (10.3)	13 (24.1)	0.089
PAIVS	15 (16.1)	6 (15.4)	9 (16.7)	0.868
Double-inlet left ventricle	13 (14.0)	6 (15.4)	7 (13.0)	0.740
ccTGA	10 (10.8)	3 (7.7)	7 (13.0)	0.512
UAVSD	5 (5.4)	3 (7.7)	2 (3.7)	0.646
Heterotaxy syndrome	11 (11.8)	4 (10.3)	7 (13.0)	0.756
Associated cardiac anomaly				
TGA	26 (28.0)	11 (28.2)	15 (27.8)	0.964
Double-outlet right ventricle	8 (8.6)	2 (5.1)	6 (11.1)	0.461
Dextrocardia	16 (17.2)	8 (20.5)	8 (14.8)	0.472
Stage II (BCPS)				
Age (months)	4.5 (3.4–5.9)	4.5 (3.2–5.7)	4.7 (3.7–6.0)	0.908
Weight (kg)	5.4 (4.8–6.0)	5.5 (4.8–6.3)	5.3 (4.8–5.9)	0.446
PAP (mmHg)	14 (12–17)	15 (12–17)	14 (12–17)	0.538
PA index	174 (138–236)	181 (140–268)	172 (135–226)	0.929
Right PA index	99(71–135)	110 (74–160)	96 (70–127)	0.413
Left PA index	78 (59–104)	78 (50–98)	78 (63–117)	0.258
LPA index/RPA index	0.86 (0.58–1.15)	0.66 (0.43–1.18)	0.90 (0.66–1.04)	0.463
Symmetry index	0.70 (0.50–0.88)	0.60 (0.43–0.79)	0.75 (0.60–0.92)	**0.026**
Reduced ventricular function	1 (1.)	1 (2.6)	0 (0.0)	0.274
AVV regurgitation ≥ moderate	8 (8.6)	1 (2.6)	7 (13.0)	0.199

ccTGA: congenitally corrected transposition of the great arteries; DS: ductal stent; PAIVS: pulmonary atresia with intact ventricular septum; SPS: systemic-to-pulmonary shunt; UAVSD: unbalanced complete atrioventricular septum defect. Bold indicates *P* < 0.05.

### Peri-procedural data at TCPC

At the cardiac catheterization before TCPC, PA index (184 vs 183 mm^2^/m^2^, *P* = 0.988) and PA symmetric index (0.62 vs 0.65, *P* = 0.546, Fig. 1B) were similar between the groups (Table 2). However, VVCs were more frequently observed in patients after DS compared to those after APS (35.9 vs 16.7%, *P* = 0.034). Operatively, median cardiopulmonary bypass (CPB) time was longer in patients after DS than after APS (73 vs 55 min, *P* = 0.011). There was no hospital death in each group. The probability of prolonged effusion for more than 7 days (59.0 vs 46.3%, *P* = 0.227), chylothorax (23.1 vs 24.1%, *P* = 0.991), and ascites needing drainage (20.5 vs 16.7%), *P* = 0.636) were similar between the groups.

**Table 2:** Perioperative variables at extracardiac TCPC

Variables	Total	DS	APS	*P*-value
*N* (%) or median (IQR)	*n* = 93	*n* = 39	*n* = 54	
Pre-TCPC variables				
PAP (mmHg)	9 (8–11)	9 (8–11)	9 (8–11)	0.376
TPG (mmHg)	4 (3–5)	4 (3–4)	4 (3–5)	0.387
LAP (mmHg)	5 (4–7)	6 (5–8)	5 (4–6)	0.070
PA index	183 (151–234)	184 (158–225)	183 (148–240)	0.988
Right PA index	110 (84–143)	113 (85–144)	107 (80–136)	0.426
Left PA index	76 (55–95)	74 (55–89)	76 (55–104)	0.581
Left to right PA index ratio	0.65 (0.41–1.07)	0.63 (0.38–1.02)	0.73 (0.49–1.10)	0.475
PA symmetry index	0.63 (0.42–0.76)	0.62 (0.40–0.72)	0.65 (0.49–0.84)	0.546
APCs	46 (49.5)	20 (51.3)	26 (48.1)	0.765
VVCs	23 (24.7)	14 (35.9)	9 (16.7)	**0.034**
Reduced ventricular function	0 (0.0)	0 (0.0)	0 (0.0)	.
AVV regurgitation ≥ moderate	4 (4.3)	0 (0.0)	3 (5.6)	0.201
Operative variables				
Age at TCPC (years)	1.9 (1.7–2.4)	1.9 (1.7–2.3)	1.8 (1.7–2.4)	0.493
Weight at TCPC (kg)	11 (10–13)	12 (11–13)	11 (10–12)	0.596
Conduit diameter (mm)				
16	2 (2.2)	1 (2.6)	1 (1.9)	0.320
18	88 (94.6)	38 (97.4)	50 (92.6)	
20	3 (3.2)	0 (0.0)	3 (5.6)	
Fenestration	12 (12.9)	7 (17.9)	4 (7.4)	0.218
CPB time (min)	63 (48–95)	73 (58–104)	55 (46–76)	**0.011**
Need for AXC	13 (14.0)	7 (17.9)	6 (11.1)	0.348
AXC time (min)	35 (17–75)	35 (20–57)	33 (15–95)	0.963
Concomitant procedures				
PA reconstruction	5 (5.4)	4 (10.3)	1 (1.9)	0.076
AVV procedure	4 (4.3)	0 (0.0)	4 (7.4)	0.082
Atrioseptecomy	4 (4.3)	2 (5.1)	2 (3.7)	0.738
Post TCPC variables				
Hospital death	0 (0)	0 (0)	0 (0)	
ICU stay (days)	5 (3–6)	5 (3–7)	5 (3–6)	0.542
HSP stay (days)	14 (12–21)	17 (13–21)	14 (12–22)	0.767
Prolonged effusion	48 (51.6)	23 (59.0)	25 (46.3)	0.227
Chylothorax	22 (23.7)	9 (23.1)	13 (24.1)	0.991
Ascites	17 (18.3)	8 (20.5)	9 (16.7)	0.636

APCs: aortopulmonary collaterals; AVV: atrioventricular valve; AXC: aortic cross-clamp; CPB: cardiopulmonary bypass; DS: ductal stent; HSP: hospital; ICU: intensive care unit; LAP: left atrial pressure; PA: pulmonary artery; PAP: pulmonary artery pressure; SPS: systemic-to-pulmonary shunt; TCPC: total cavopulmonary connection; TPG: transpulmonary gradient; VVCs: venovenous collaterals. Bold indicates *P* < 0.05.

### Follow-up data

**Figure 2:**
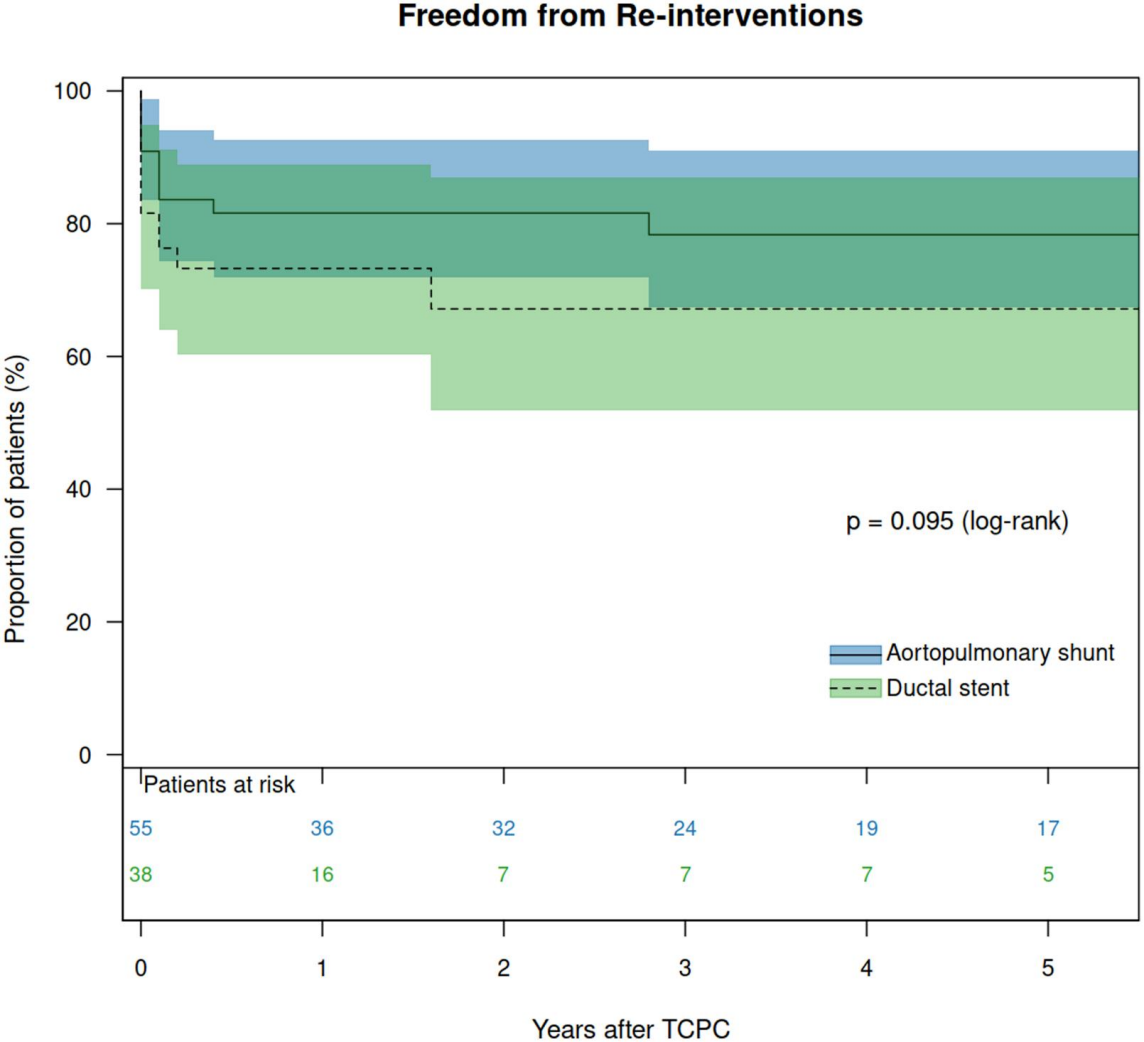
Freedom from PA reinterventions after TCPC in patients after DS and those after APS.

**Figure 3:**
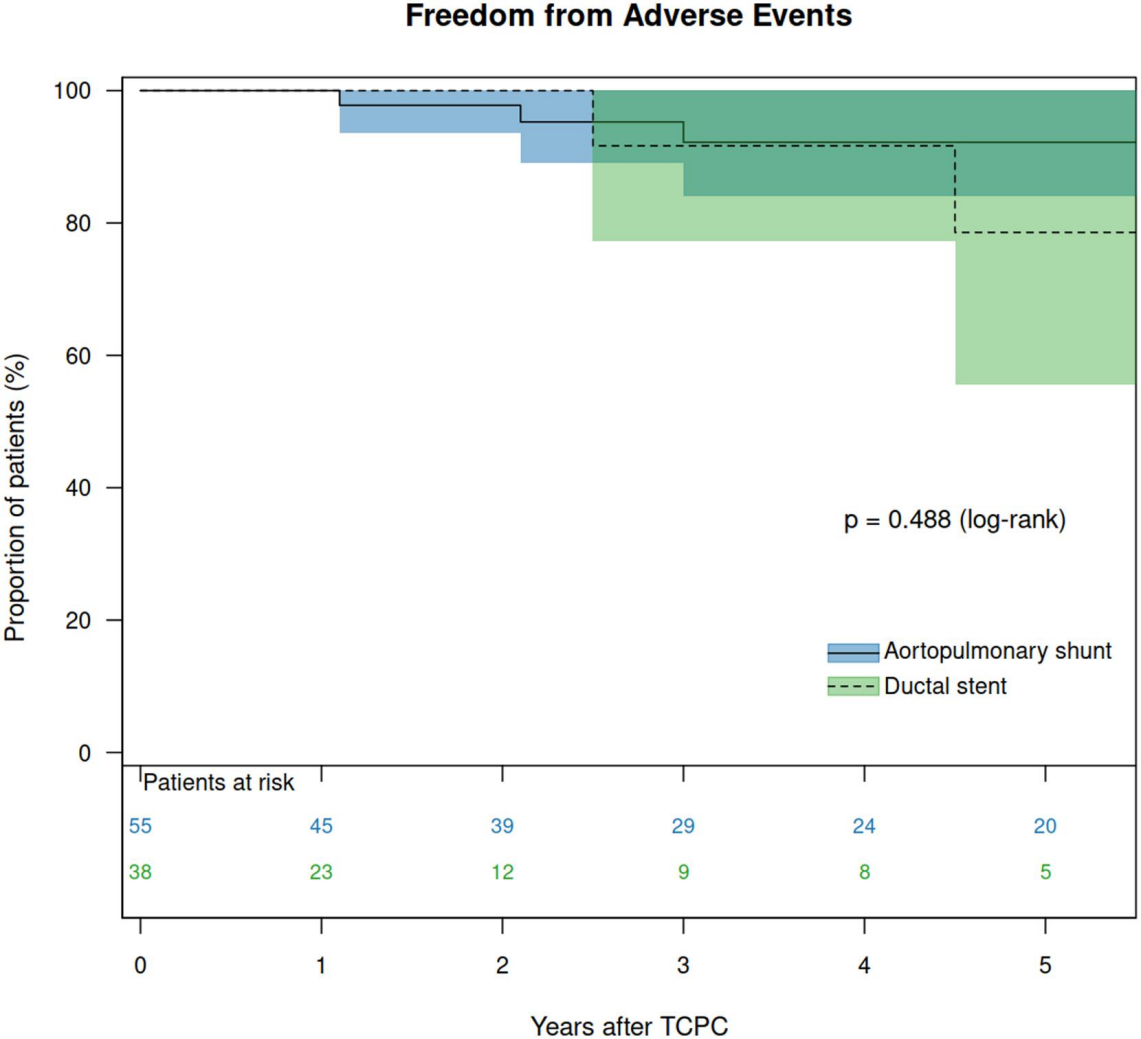
Freedom from adverse events after TCPC in patients after DS and those after APS.

**Figure 4:**
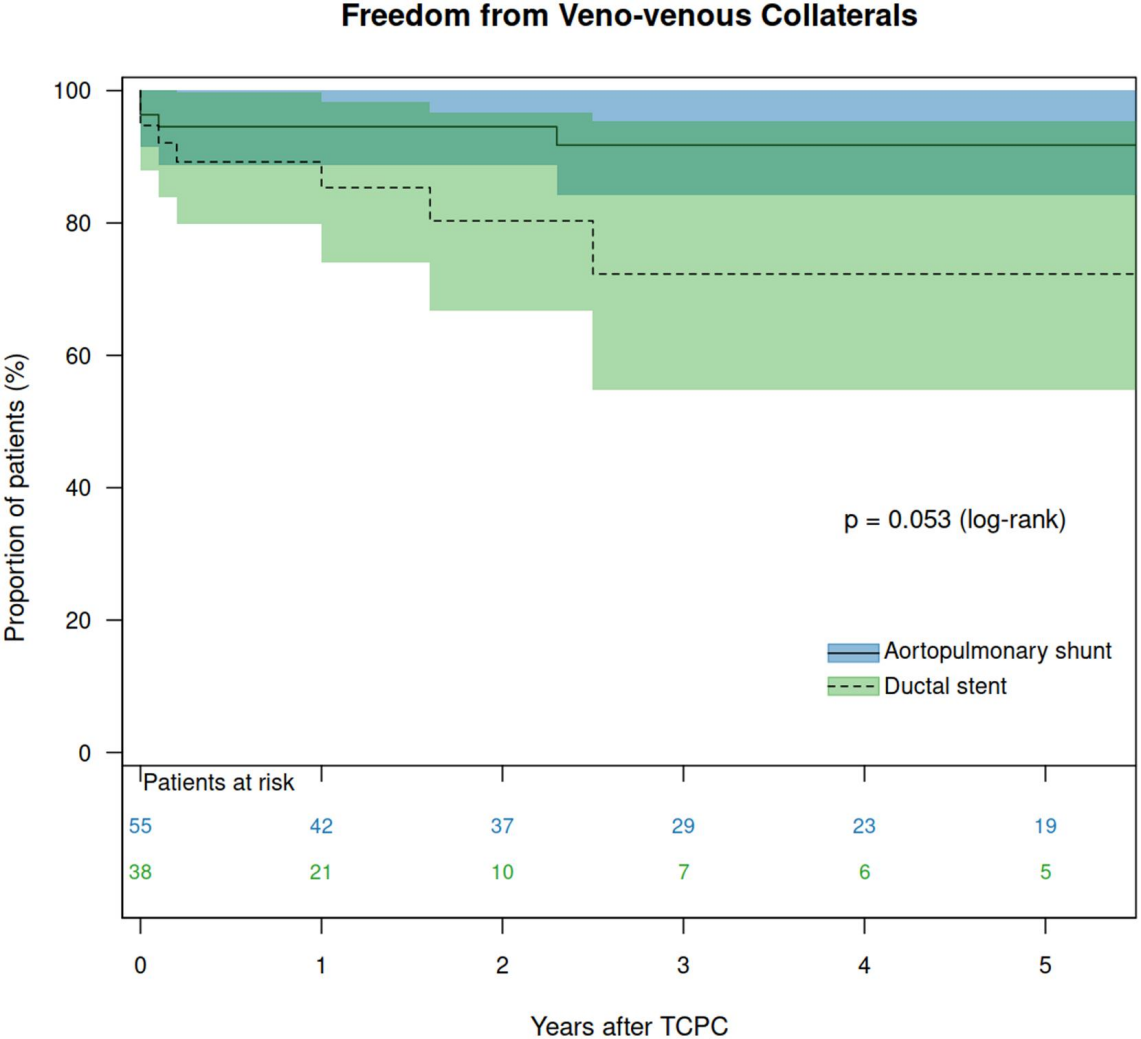
Freedom from the development of venovenous collaterals after TCPC in patients after DS and those after APS.

The median potential follow-up time after TCPC, calculated using the reverse Kaplan–Meier method, was 2.5 (95% CI: 1.8–3.5) years. The overall CCI was 0.313, and the SPT method yielded 0.317. For the DS group, these values were 0.348 and 0.352, respectively, compared to 0.399 and 0.399 for the APS group. The median follow-up duration was 1.7 years for the DS group and 3.9 years for the APS group. There was one late death in the DS group. There was no heart transplantation in either the DS or the APS group. Therefore, transplant-free survival 5 years after TCPC was 85.7% in the DS group and 100% in the APS group (*P* = 0.070, Supplementary Fig. 2). Catheter interventions for PA were performed in 22 patients (11 in the DS group and 11 in the APS group). Freedom from reintervention 5 years after TCPC was 66.6% in the DS group and 82.0% in the APS groups (*P* = 0.095, Fig. 2). High pulmonary pressure at TCPC (hazard ratio [HR]: 1.284, *P* = 0.020), high left atrial pressure (HR: 1.351, *P* = 0.027), low PA index at BCPS (HR: 0.992, *P* = 0.043), low left PA index at TCPC (HR: 0.981, *P* = 0.025) and low symmetry index at TCPC (HR: 0.026, *P* = 0.004) were identified as risk factors for reinterventions after TCPC using univariable model (Supplementary Table 1). Multivariable analysis revealed high left atrial pressure (HR: 1.351, *P* = 0.027) as an independent risk for reinterventions after TCPC. Adverse events were observed in five patients (two in the DS group and three in the APS group), including one death and four PLE. The details of the patients are shown in Table 3. All patients with adverse events had left PA stenosis, four of them had stent implantations in the left PA, and the remaining patient had surgical left PA reconstruction concomitant with BCPS. Freedom from adverse events 5 years after TCPC was 78.6% in the DS group and 92.2% in the APS group (*P* = 0.488, Fig. 3). VVCs following TCPC were found in 11 patients (seven patients after DS and four after APS). Freedom from the development of VVCs was not significantly different between the DS group and the APS group (*P* = 0.053, Fig. 4).

**Table 3:** Patients with adverse events

Pt	Main diagnosis	Heterotaxy	Stage I			Stage II		Interstage II-III		Stage III and follow-up			
			S1P	Age (d)	Events after S1P	BCPS	Age (m)	PA intervention	Interval	Age (m)	Events after TCPC	Adverse events	Outcome
1	DORV, hypoLV, pulmonary stenosis	no	BTS 3.5	10	ECMO, thrombus in PA	LPA patch	4	no		20	APCs closure, VVCs closure	PLE	alive
2	UAVSD, hypo LV, pulmonary atresia	yes	DS 3.5	1	no	PA patch	3	LPA stent, VVCs closure	17 d	17	LPA stent dilatation, VVCs closure	Sudden death	death
3	SV, pulmonary stenosis	no	BTS 3.5	8	no	LPA patch	3	APCs closure	13 m	17	LPA stent implantation	PLE	alive
4	DILV, TGA, pulmonary stenosis	no	DS 3.5	14	no	no	4	LPA stent	10 d	23	LPA stent dilatation, APCs closure,	PLE	alive
5	CAVSD, TGA, pulmonary atresia	yes	BTS 3.5	4	no	LPA patch	3	no		22	LPA stent implantation	PLE	alive

## DISCUSSION

### DS versus APS as an initial palliation

Nowadays, DS is performed by many centres to provide initial palliation for neonates with various types of congenital heart disease who have PDA-dependent pulmonary circulation [1–8]. As the technical feasibility is improving, DS may be considered for all patients with single ventricle and PDA-dependent pulmonary circulation. One important reason is the high mortality after neonatal APS in single ventricle patients, ranging from 6% to 18% in previous studies [9–12]. In our centre, the indication for surgical APS has become limited for neonates in whom the procedural success of the interventional approach may not be reached or after a failed attempt of DS. This includes neonates with closed PDAs, large PDAs of more than 4 mm diameter or strongly meandered PDAs. Fortunately, low body weight below 2.5 kg, a strong risk factor for surgical APS, does not increase the risk for DS [5]. The great advantage of DS over APS is a shorter length of intensive care unit (ICU) and hospital stays. Our previous study demonstrated a median 1-day stay in the ICU after DS, compared to 7 days after APS [8], and other studies showed similar results [3–7]. On the contrary, the incidence of acute complications such as stent dislocation or stent dysfunction was higher in DS than in APS. Stent dislocation and dysfunction are constantly observed in challenging cases and surgical rescue therapy through urgent APS is needed. However, Vida *et al.* demonstrated that operations after DS are safe and low-risk, despite the fact that the presence of DS frequently required additional surgical manoeuvres on the PA and postoperative re-interventions [21]. As for mortality after the initial palliation, our previous study demonstrated no significant difference in hospital mortality or interstage mortality between the initial palliation and the second-stage BCPS [8]. The cumulative incidence of the rate of Fontan completion in this study was not significantly different. As for the PA interventions before TCPC, the prevalence of PA reconstruction at BCPS was similar in patients after DS and those after APS. However, interstage PA interventions between BCPS and TCPC were more frequently performed in patients after DS than after APS [22]. Because of the extreme shortage of donations for infants and small children in Germany, no patient underwent heart transplantation during or after the staged Fontan palliation, and it is an issue to be solved in the future. Tachyarrhythmia after TCPC was rare in our cohort, as described in our previous study [23]. Therefore, we did not include tachyarrhythmia in the adverse events.

Our institution has a policy to perform TCPC when patients are 18 months of age and have a weight of 10 kg. Our previous study demonstrated that TCPC can be performed before or at 18 months of age without the expense of increased morbidity or mortality [24]. Therefore, the previous interstage interventions for PA are not considered to delay the timing of TCPC.

### Impact of DS on the development of PA

Glatz *et al.* and Santoro *et al.* showed balanced catch-up growth of PA in patients after PDA stenting [5, 13–15]. Meanwhile, Helal *et al.* reported that DS patients had a higher rate of stent thrombosis and inter-stage re-interventions [16]. Our data showed that the PA symmetry index at the time of BCPS was lower in patients after DS than those after APS. Therefore, it is of note that the PA symmetry index was similar at the time of TCPC between DS and APS. It might be due to the inter-stage PA interventions between BCPS and TCPC. Our previous study demonstrated that DS patients had a higher rate of inter-stage reintervention during the staged Fontan palliation [8]. PA index, right PA index and left PA index were also similar between the groups at the time of TCPC. Interestingly, the incidence of VVCs was higher in patients after DS compared to those after APS. Our previous study demonstrated that VVCs after TCPC were associated with a high prevalence of plastic bronchitis [25], and VVCs between BCPS and TCPS were induced by an elevated PAP and also by more previous palliations (not published data). Therefore, we think that the development of VVCs is caused by sub-optimal condition of the cavopulmonary circulation, such as high PA, small PA size, or high pulmonary vascular resistance.

### Impact of DS on the outcome after TCPC

There was no difference in in-hospital morbidity following TCPC in patients after DS and APS regarding prolonged pleural effusion, chylothorax and ascites. In the follow-up, survival and freedom from adverse events were similar between the groups. It is of note that patients following DS required longer CPB time than those following APS. It might be caused by the relatively frequent PA reconstruction at TCPC in patients following DS (10%) compared to those following APS (2%). Previous stent implantation might cause the adhesion of the PA with the surrounding tissues, and it might be another cause of longer CPB in patients following DS. During the follow-up, more than 30% of the patients following DS required re-interventions within the first 5 years after TCPC, whereas less than 20% required re-interventions following APS, although there was no statistical difference (*P* = 0.095). Furthermore, more than 25% of the patients following DS developed VVCs within the first 5 years after TCPC, whereas less than 10% developed VVCs following APS. Again, there was no statistical difference (*P* = 0.053). The number of patients was small and the follow-up period was short in this study. Further studies are mandatory to determine the impact of DS on late outcomes after the Fontan procedure. We suspect that a relatively small left PA and repeated interventions for left PA in the DS patients might affect the long-term outcomes after TCPC.

### Future prospective

If DS is successful, it reduces ICU and hospital stays and, consequentially, treatment costs. Until now, DS is not always successful or feasible, and some patients still need surgical APS. The evolving techniques and newly developed stent products might improve early results after DS in neonates and the results in terms of PA development up to the Fontan stage and beyond.

### Limitations

This study has clear drawbacks due to its retrospective nature and single-centre analysis with its inherent biases. The nonrandomized nature of this study, either DS or APS, limits the interpretation of the results significantly. A small number of patients does not have the power to detect clinically relevant differences between the groups or to perform statistical adjustments such as stratification by key demographics or clinical characteristics. According to a retrospective sample size estimate, a minimum of 130 patients would have been required to detect a significant difference in the freedom from reintervention between the two groups. A short follow-up period could not identify major post-Fontan complications that could appear in the long term. The decision-making process has changed during the study period, adding to possible biases. The long-term outcomes, including survival and neurological outcomes, were not evaluated. Patient selection bias was an obvious limitation because our approach has generally been to perform DS in patients with impaired status prior to the intervention to avoid potential adverse events following surgery.

## CONCLUSIONS

In this pilot study with a limited number of patients, we did not find a significant difference in outcomes after the Fontan procedure between patients with single ventricle and PDA-dependent pulmonary blood flow and between patients after initial DS and those after surgical APS. However, DS patients tended to require more re-interventions than those who underwent APS. Although this preliminary study supports both DS and APS as viable strategies for managing patients with single ventricle and PDA-dependent pulmonary blood flow, further randomized studies, including large-scale cohorts and long-term follow-up, are mandatory to confirm the safety and efficacy of DS in the long-term after the Fontan completion.

## Supplementary Material

ivaf118_Supplementary_Data

## Data Availability

The data that support the findings of this study are available from the corresponding author upon reasonable request.

## Notes

Presented at the 38th Annual Meeting of the European Association for Cardio-Thoracic Surgery, Lisbon, Portugal, October 9–12, 2024.

## ABBREVIATIONS

APS: Aortopulmonary shunt
BCPS: Bidirectional cavopulmonary shunt
CCI: Clark’s Completeness Index
CPB: Cardiopulmonary bypass
DS: Ductal stenting
ICU: Intensive care unit
IQR: Interquartile range
PA: Pulmonary artery
PDA: Patent ductus arteriosus
PLE: Protein-losing enteropathy
SPT: Simplified Person-Time
TCPC: Total cavopulmonary connection
VVCs: Venovenous collaterals
