# Supplementary material for: Comparison of outcomes following the Fontan procedure between patients with previous ductus stent and aortopulmonary shunt
Source: Interdiscip Cardiovasc Thorac Surg. 2025 May 21;40(6):ivaf118. doi: 10.1093/icvts/ivaf118 (PMC12145171; doi:10.1093/icvts/ivaf118)
Supplement: ivaf118_Supplementary_Data [file ivaf118_supplementary_data.zip › Supplementary Tables19012025.docx]

**Supplementary Tables**

Supplementary Table S1

| Table S1. Risk factors for reinterventions after TCPC | | |  |  |  |  |  |
| --- | --- | --- | --- | --- | --- | --- | --- |
| Variables |  | Univariable |  |  |  | Multivariable |  |
|  | HR | 95% CI | p-value |  | HR | 95% CI | p-value |
| DS | 2.027 | 0.875-4.918 | 0.118 |  |  |  |  |
| BCPS PAP | 0.916 | 0.769-1.092 | 0.329 |  |  |  |  |
| BCPS TPG | 0.965 | 0.793-1.174 | 0.721 |  |  |  |  |
| BCPS LAP | 1.094 | 0.972-1.232 | 0.135 |  |  |  |  |
| TCPC PAP | 1.284 | 1.041-1.584 | **0.020** |  |  |  |  |
| TCPC TPG | 0.960 | 0.713-1.292 | 0.786 |  |  |  |  |
| TCPC LAP | 1.336 | 1.124-1.587 | **<0.001** |  | 1.351 | 1.034-1.765 | **0.027** |
| BCPS PA index | 0.992 | 0.985-1.000 | **0.043** |  |  |  |  |
| BCPS rPA index | 0.994 | 0.984-1.004 | 0.234 |  |  |  |  |
| BCPS lPA index | 0.987 | 0.974-1.002 | 0.080 |  |  |  |  |
| BCPS left to right ratio | 1.246 | 0.574-2.705 | 0.579 |  |  |  |  |
| BCPS symmetry index | 0.471 | 0.045-4.925 | 0.529 |  |  |  |  |
| TCPC PA index | 0.994 | 0.986-1.002 | 0.159 |  |  |  |  |
| TCPC rPA index | 1.002 | 0.992-1.012 | 0.699 |  |  |  |  |
| TCPC lPA index | 0.981 | 0.964-0.998 | **0.025** |  |  |  |  |
| TCPC left to right ratio | 0.466 | 0.123-1.767 | 0.261 |  |  |  |  |
| TCPC symmetry index | 0.026 | 0.002-0.308 | **0.004** |  |  |  |  |
| APCs before TCPC | 0.955 | 0.368-2.476 | 0.924 |  |  |  |  |
| VVCs before TCPC | 0.986 | 0.289-3.363 | 0.982 |  |  |  |  |
| Age at TCPC | 1.188 | 0.773-1.825 | 0.431 |  |  |  |  |
| Tricuspid atresia | 0.858 | 0.287-2.567 | 0.784 |  |  |  |  |
| DILV | 1.215 | 0.356-4.148 | 0.756 |  |  |  |  |
| PAIVS | 0.908 | 0.266-3.100 | 0.878 |  |  |  |  |
| UAVSD | 1.998 | 0.463-8.614 | 0.353 |  |  |  |  |
| UVH | 0.477 | 0.111-2.057 | 0.321 |  |  |  |  |
| ccTGA | 0.040 | 0.000-13.651 | 0.280 |  |  |  |  |
| TGA | 0.657 | 0.219-1.964 | 0.452 |  |  |  |  |
| DORV | 0.043 | 0.000-32.720 | 0.352 |  |  |  |  |
| Dextrocardia | 1.296 | 0.433-3.884 | 0.643 |  |  |  |  |
| Heterotaxy | 1.277 | 0.374-4.363 | 0.696 |  |  |  |  |
