# Supplementary figures and images for: Comparison of outcomes following the Fontan procedure between patients with previous ductus stent and aortopulmonary shunt
Source: Interdiscip Cardiovasc Thorac Surg. 2025 May 21;40(6):ivaf118. doi: 10.1093/icvts/ivaf118 (PMC12145171; doi:10.1093/icvts/ivaf118)

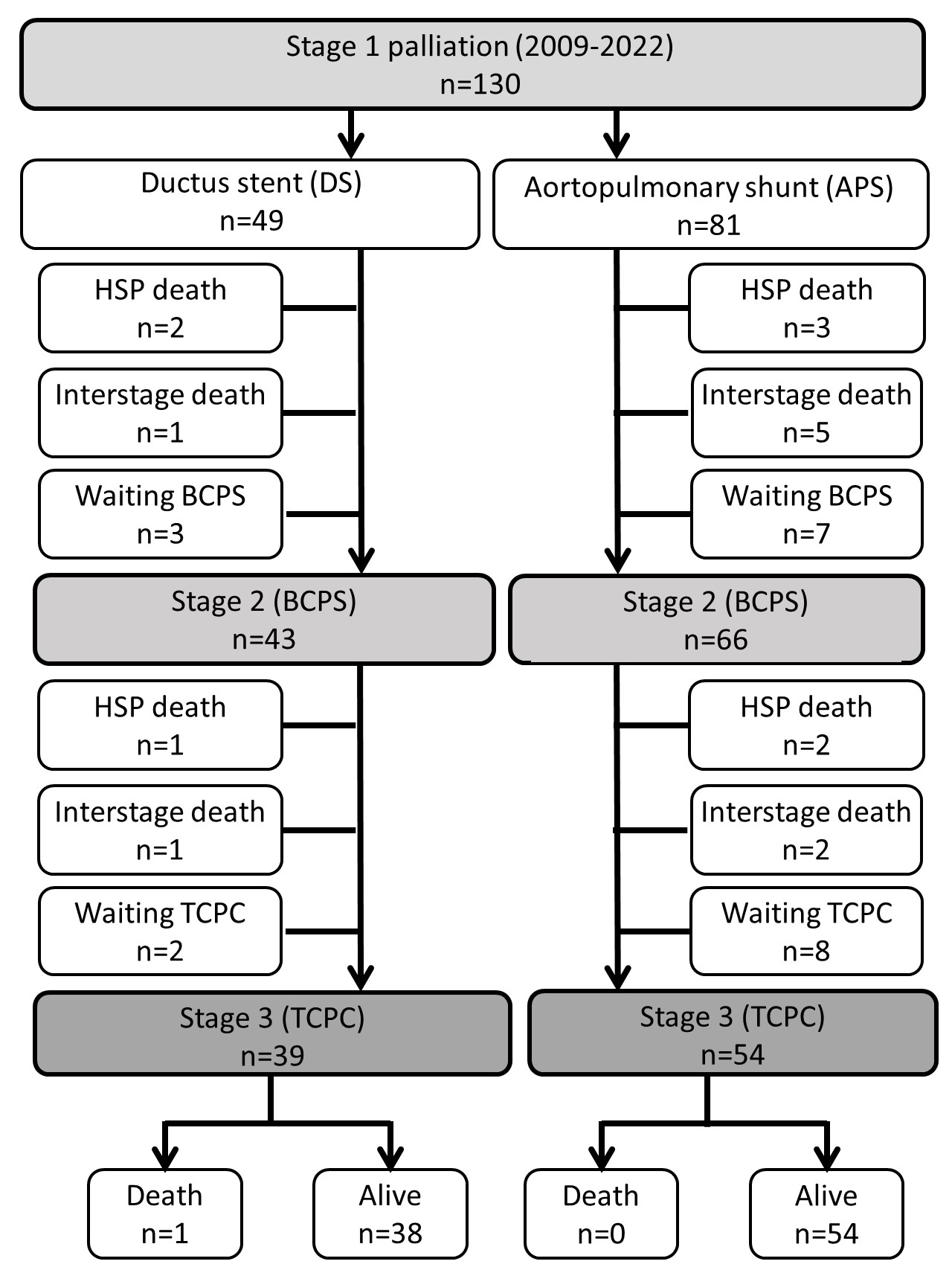

Supplement: ivaf118_Supplementary_Data [file ivaf118_supplementary_data.zip › SupplFigureS1Flowchartf04032025.jpg]
